# Using the longest run subsequence problem within homology-based scaffolding

**DOI:** 10.1186/s13015-021-00191-8

**Published:** 2021-06-28

**Authors:** Sven Schrinner, Manish Goel, Michael Wulfert, Philipp Spohr, Korbinian Schneeberger, Gunnar W. Klau

**Affiliations:** 1 Algorithmic Bioinformatics, Heinrich Heine University Düsseldorf, Düsseldorf, Germany; 2 Heinrich Heine University Düsseldorf, Düsseldorf, Germany; 3 Cluster of Excellence on Plant Sciences (CEPLAS), Heinrich Heine University Düsseldorf, Düsseldorf, Germany; 4 Max Planck Institute for Plant Breeding Research, Cologne, Germany; 5 Faculty of Biology, LMU Munich, Großhaderner Str. 2, 82152 Planegg-Martinsried, Germany

**Keywords:** Alignment, Assembly, String algorithm, Longest subsequence

## Abstract

Genome assembly is one of the most important problems in computational genomics. Here, we suggest addressing an issue that arises in homology-based scaffolding, that is, when linking and ordering contigs to obtain larger pseudo-chromosomes by means of a second incomplete assembly of a related species. The idea is to use alignments of binned regions in one contig to find the most homologous contig in the other assembly. We show that ordering the contigs of the other assembly can be expressed by a new string problem, the longest run subsequence problem (LRS). We show that LRS is NP-hard and present reduction rules and two algorithmic approaches that, together, are able to solve large instances of LRS to provable optimality. All data used in the experiments as well as our source code are freely available. We demonstrate its usefulness within an existing larger scaffolding approach by solving realistic instances resulting from partial *Arabidopsis thaliana* assemblies in short computation time.

## Introduction

Genome assembly from sequencing reads enables the analysis of an organism at its genome level and is one of the most important problems in computational genomics. The first step is usually to assemble the reads based on overlap- or *k*-mer-based approaches to create contigs, which then need to be put into correct order and orientation in a scaffolding phase to generate the final assembly. The presence of a high-quality chromosome-level reference genome of the same species can significantly simplify assembly generation as it can be used as a template to order these contigs [1, 2]. However, for many species, such a reference genome is not available.

There are two commonly used approaches for scaffolding. First, different types of maps provide anchors for the contigs in the genome. These could be, for example, genetic maps, physical maps or cytological maps providing markers with known positions in the genome and known distances between each other [3]. The other approach is to use long-range genomic information to link multiple contigs and put them into correct order and orientation. Prominent examples are linked barcoded reads like 10X sequencing [4], Hi-C data based on chromatin conformation capture [5] and optical mapping [6].

**Fig. 1:**
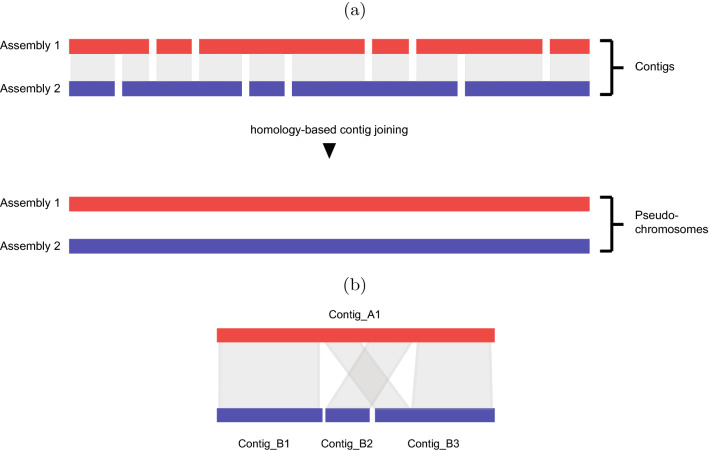
Homology-based scaffolding. **a** Independent initial assemblies (contigs), which are joined into pseudo-chromosomes by using homologies between contigs for scaffolding. **b** Alignments between contigs from different samples. A1 determines the order of B1, B2 and B3

Yet another way for contig scaffolding is to use two or more incomplete assemblies from closely related samples [7]. Regions of unconnected contigs for one sample might be connected with the help of another, related sample, e.g., a genome assembly of an individual of the same species, providing an overall gain in information for both samples. Local similarities between contigs from different samples can be used to align and order them. Ideally, this leads to long chromosome-like sequences called pseudo-chromosomes, where the contigs of different samples are aligned like shingles next to each other, as illustrated in Fig. 1(a). Note that this setting differs from the problem of assembly reconciliation [8], where the task is to build a consensus assembly from two or multiple input assemblies from the same species but which does not make use of homology information from different species.

Note that structural rearrangements such as translocations or inversions and repeat regions between genomes can result in non-sequential and non-unique mappings within contigs and can thus lead to misleading connections between contigs. These events need to be considered when finding homologous contigs as shown in Fig. 1(b).

In the simplest setting of two incomplete assemblies we are given two sets of contigs $A = \{A_1, \ldots , A_l\}$ and $B = \{B_1, \ldots , B_m\}$ computed from two different samples. As already stated, the contigs are not ordered with respect to genome positions, and it is this order we rather want to compute. More precisely, we aim at inferring the most likely order from between-sample overlaps among the contigs.

Assuming we want to order the contigs in *B*, we first map the contigs $A_1, \ldots , A_l$ against the contigs of *B*, divide every contig $A_i$ of *A* into smaller, equally sized chunks, called *bins* and determine the best matching contig in *B* for every bin after. If $A_i$ actually overlaps with multiple contigs in *B*, we should be able to partition $A_i$ into smaller parts based on mapping the bins to different contigs in *B*. However, sequencing or mapping errors as well as mutations between the samples can cause some bins to map onto a “wrong” contig, i.e., a contig belonging to a different area than the bin. Therefore a method to find the best partition of $A_i$ needs to distinguish between actual transitions from one *B*-contig to another and noise introduced by errors or mutations.

**Fig. 2:**
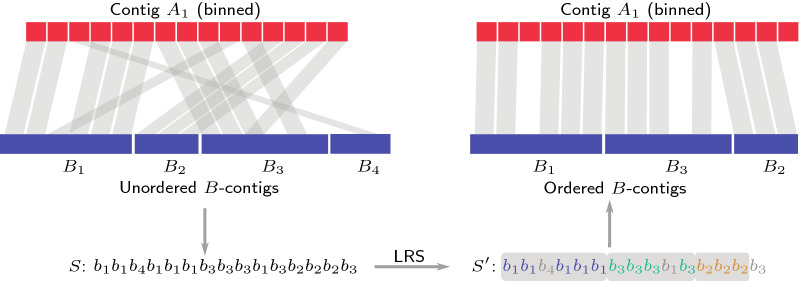
Processing of a single contig $A_1$. The bins are matched against all contigs of another sample *B*. Solving Longest Run Subsequence (LRS) on the corresponding string *S*, yields a maximal subsequence with at most one run for every contig. This induces an optimal order for a subset of *B*-contigs

Figure 2 illustrates the different steps in solving this problem. Starting from a binned contig from *A*, here $A_1$ for illustration, and its mapping preferences to the unordered contigs in *B*, we reformulate this ordering problem as a string problem. In essence, we want to find the longest subsequence of the input string of mapping preferences that consists only of consecutive runs of contigs from *B* where each such run may occur at most once. This subsequence corresponds to an ordering of the contigs in *B*, which can be transferred to the original problem.

In this paper we formalize this process and introduce the *Longest Run Subsequence* problem (LRS). We show that LRS is NP-hard. Nevertheless, we want to solve large instances of LRS to provable optimality in reasonable running time and therefore present a number of reduction rules and two algorithms based on integer linear programming and dynamic programming, respectively. We evaluate both approaches on synthetic instances and find that they show complementary strengths regarding the number of runs and the alphabet size. We also test our approaches on realistic instances within the initial scaffolding phase of the SyRI package [7]. The test instances occurred during assembly of *Arabidopsis thaliana* samples and could not be solved by a brute-force method. We show that all those instances can be solved within short computation time. Our code and all data used in the experiments are freely available at https://github.com/AlBi-HHU/longest-run-subsequence. The software can also be installed with pip as a module from https://pypi.org/project/longestrunsubsequence/ or as “longestrunsubsequence” from bioconda and can thus easily be used within larger scaffolding packages.

## Problem formulation

A string $S = s_1, \ldots , s_m$ is a sequence over characters from a finite alphabet $\Sigma$. A subsequence of *S* is a sequence $s_{i_1}, \ldots , s_{i_k}$, such that $1 \le i_1< i_2< \ldots < i_k \le m$. We denote the *substring* $s_i, \ldots , s_j$ of *S* as *S*[*i*, *j*] and *k* consecutive occurrences of a character $\sigma$ inside a string *S* as $\sigma ^k$ and call it a *run*. Let $\sigma (r)$ be the character of the run *r* and $L(r)$ its length. By summarizing the characters of *S* into maximally long runs, *S* can be represented as a unique sequence of runs $r_1, \ldots , r_n = \sigma (r_1)^{L(r_1)} \ldots \sigma (r_n)^{L(r_n)}$. For every $\sigma \in \Sigma$ we define $P_{\sigma }(i)$ as the index of the last run before $r_i$ containing $\sigma$ in *S* (0 if it does not occur). As an example, the string from Fig. 2 can be compressed to ${b_1}^2 {b_4}^3 {b_1}^3 {b_3}^3 {b_1}^1 {b_3}^1 {b_2}^3 {b_3}^1$ with $P_{b_1}(4) = 3$, $P_{b_1}(3) = 1$ and $P_{b_4}(1) = 0$.

We propose to model the optimal partition of a single contig as a string optimization problem. Formally, we use the contigs from set *B* as the alphabet, that is $\Sigma = \{b_1, \ldots , b_m\}$ and write the contig $A_i$ as a string $S = b_{i_1} \ldots b_{i_m}$ over $\Sigma$ by replacing the bins of $A_i$ with the corresponding character of the best match from *B*. On the one hand, we want every single bin to be assigned to its preferred contig in *B*, but, on the other, we also want a simple partition, which is not skewed by wrong mappings of single bins. We therefore restrict valid partitions of $A_i$ to contain at most one contiguous part for every contig in *B*. This prevents large parts to be interrupted by single mismatching bins, at the cost of not being able to capture short-ranged translocations as seen in Fig. 1(b). A partition can be represented as a subsequence $S'$ of the string *S*, which only contains at most one run for every $\sigma \in \Sigma$. The runs in $S'$ are the parts corresponding to one *B*-contig each, while the dropped characters from *S* are bins in conflict with $S'$. Finding the best partition can thus be stated as the following optimization problem:

### Problem 1

**(Longest Run Subsequence, LRS)** Given an alphabet $\Sigma = \{\sigma _1, \ldots , \sigma _{|\Sigma |}\}$ and a string $s=s_1, \ldots , s_m$ with $s_i \in \Sigma$, find a longest subsequence $S' = s'_1, \ldots , s'_k$ of *S*, such that $S'$ contains at most one run for every $\sigma \in \Sigma$. That is, for every pair of positions *i* and *j* with $i<j$, it holds that

$$\begin{aligned}s'_i = s'_j \,\Rightarrow \, s'_l = s'_i \quad \text {for all } i< l < j.\end{aligned}$$

We denote the length of an optimal LRS solution for *S* with $\text {LRS}\left( S\right)$. Since we want to maximize the length of the run subsequence, it is always beneficial to either completely add or completely remove a run of *S*. Once a character $s_i \in \Sigma$ from a run $s_i^k$ is added to $s'$, there can never be any other occurrence of $s_i$ outside this run. Thus, the entire run must be added to $s'$ to achieve maximum length. We will therefore mainly refer to runs instead of single characters.

## Complexity

In this section we prove hardness of the Longest Run Subsequence problem. More precisely, we show that dLRS, the decision version of the problem is NP-complete. An instance of dLRS is given by a tuple (*S*, *k*) and consists in answering the question whether *S* has a longest run subsequence of length at least *k*.

### Theorem 1

*dLRS is NP-complete.*

### *Proof*

It is easy to see that dLRS is in NP, because it can be checked in polynomial time whether a string $s'$ is a solution, that is, $s'$ is a run subsequence and $|s'| \ge k$.

To prove NP-hardness, we reduce from the Linear Ordering Problem (LOP), which has been shown to be NP-hard [9]. LOP takes a complete directed graph with edge weights and no self-loops as input and looks for an ordering among the vertices, such that the total weights of edges following this order (i.e., edges leading from lower ordered vertices to higher ordered vertices) is maximized.

We show that dLOP, the decision problem of LOP, that is, the question whether a vertex ordering exists whose weight is at least a given threshold, can be polynomially reduced to dLRS. Let $G=(V,E)$ be a complete digraph with $|V |=n$. We denote the weight of $(v_i, v_j) \in E$ with $w_{ij}$ and the sum of all weights of *G* as $w_{\text {sum}}$. Without loss of generality we can assume that all edge weights are positive: The number of edges following a linear order is fixed, so adding a sufficiently large offset to all weights only adds a fixed value to any solution without changing the core problem. This allows us to characterize LOP as finding an acyclic subgraph $G'$ with maximum weight, because the non-negativity of the weights always forces either $(v_i, v_j)$ or $(v_j, v_i)$ to be in $G'$ for every pair of vertices $v_i, v_j \in V$.

The proof consists of two parts. First, we show how to transform *G* into a string *S*. Second, we show that *G* has a LOP solution of weight *k* if and only if *S* has a LRS of size

1 $$\begin{aligned} f_G(k) := (n-1) \cdot M + \frac{n(n-1)(n-2)}{3} \cdot M' + n(n-1) \cdot w_{\text {sum}} + 2k \end{aligned}$$

with $$M':= 4n^2 \cdot w_{\text {sum}}$$ and $$M := M' \cdot n^3$$.

For the transformation, we define $\Sigma$ using three different types of characters:

1. Separators $\$_{i}$ for every vertex $v_i \in V$.
2. Edge signs $E_{\{i,j\}}$ for every pair $v_i, v_j \in V$. Note that $E_{\{i,j\}} = E_{\{j,i\}}$.
3. Triangle signs $\Delta _{(i,j,k)}$ for every triangle in *G*. Note that triangles between three vertices have an orientation and can be rotated. Therefore $\Delta _{(i,j,k)} = \Delta _{(j,k,i)} = \Delta _{(k,i,j)} \ne \Delta _{(i,k,j)} = \Delta _{(k,j,i)} = \Delta _{(j,i,k)}$.

On the highest level the string *S* is constructed as shown in Equation 2. It consists of one large block per vertex, each of them separated by a run of the associated separation sign of length *M*.

2 $$\begin{aligned} S = \underbrace{\overbrace{\text {[EB]}_{1,2}}^{\begin{array}{c} \text {edge block} \\ \text {for }(v_1,v_2) \end{array}} \text {[EB]}_{1,3} \ldots \text {[EB]}_{1,n}}_{\text {vertex block for }v_1} \$_{1}^M \text {[EB]}_{2,1} \ldots \text {[EB]}_{2,n} \$_{2}^M \ldots \$_{n-1}^M \text {[EB]}_{n,1} \ldots \text {[EB]}_{n,n-1} \end{aligned}$$

Each vertex block consists of a series of *edge blocks* (EB), which we define as follows:

3 $$\begin{aligned} \text {[EB]}_{i,j} = E_{\{i,j\}}^{w_{ij}+w_{\text {sum}}} \quad \Delta _{(i,j,1)}^{M'} \ldots \Delta _{(i,j,n)}^{M'} \quad E_{\{i,j\}}^{w_{ij}+w_{\text {sum}}} \end{aligned}$$

In the same way as the *i*-th vertex block is associated with vertex $v_i$, the edge substrings in it are associated with the outgoing edges of $v_i$. Note that there is one EB missing in every vertex block, as self-loops are not allowed. Finally, $\text {[EB]}_{i,j}$ contains all triangle signs for triangles, in which $(v_i, v_j)$ occurs, i.e., $\{\Delta _{(i,j,k)} \mid 1 \le k \le n, k \ne i, k \ne j\}$, which, for the sake of notation, is written as $\Delta _{(i,j,1)}^{M'} \ldots \Delta _{(i,j,n)}^{M'}$ in Eq. 3. The triangle signs are padded by edge signs for $(v_i, v_j)$. Every edge sign $E_{\{i,j\}}$ occurs only in the two edge blocks $\text {[EB]}_{i,j}$ and $\text {[EB]}_{j,i}$. The length of the edge sign runs depends on the weight of the corresponding edge (in either direction), rewarding the higher weighted edge. We also add $w_{\text {sum}}$ to the length of every edge sign run $E_{\{i,j\}}$.

As for the numbers *M* and $M'$, the latter is chosen to be larger than the combined length of all edge sign runs. This makes a single triangle sign run more profitable than any selection of edge sign runs. In the same manner, *M* is chosen to be larger than all triangle sign runs combined.

Using this construction, a valid solution $G'=(V,E')$ for a dLOP instance (*G*, *k*), i.e., an acyclic subgraph of *G* with total weight of at least *k*, can be transformed into a valid solution for a dLRS instance $(S, f_G(k))$. First, all separation runs are selected, yielding a total length of $(n-1) \cdot M$. Second, for every edge in $E'$, all edge signs in the corresponding edge blocks are selected. Since $|E' | = \frac{n(n-1)}{2}$, this adds at least $2 \cdot \left( \frac{n(n-1)}{2} \cdot w_{\text {sum}} +k \right)$ characters to the solution. Finally, $G'$ is acyclic, so for every triangle in *G*, there is at least one edge missing in $G'$. Thus, by construction of *S*, one run can be selected for every triangle sign without interfering with the edge sign runs, adding the missing $\frac{n(n-1)(n-2)}{3} \cdot M'$ characters.

Given a solution $S'$ for the dLRS instance $(S, f_G(k))$, we show how to obtain a subgraph $G'$ of total weight at least *k* for the original dLOP instance. The subsequence $S'$ must contain all separation runs and a run for every triangle sign, because without all separation and triangle signs selected at some place, it is (by choice of *M* and $M'$) impossible to reach length $f_G(k)$ for any *k*. Therefore every selected edge sign run belongs to a single edge block of a solution of dLRS. The idea is that the choice of selecting $E_{\{i,j\}}$ either in $\text {[EB]}_{i,j}$ or $\text {[EB]}_{j,i}$ corresponds to the choice of having either (*i*, *j*) or (*j*, *i*) in the DAG $G'$ for the original LOP. Since we added $w_{\text {sum}}$ to the length of every edge sign run and there are only $\frac{n(n-1)}{2}$ edge signs in total (with *n* being the number of vertices in *G*), $S'$ must contain both runs inside an edge block, in order to reach length $n(n-1) \cdot w_{\text {sum}}$ (the third summand in $f_G(k)$). Thus, either edge signs or triangle signs may be selected inside an edge block, but not both. $G'$ is finally obtained by selecting an edge *e* if and only if the edge sign runs in the corresponding edge block are selected. This yields $\frac{n(n-1)}{2}$ edges with a total weight of at least *k*. For every vertex pair $v_i, v_j$, exactly one of the edges $(v_i,v_j)$ and $(v_j,v_i)$ is selected, because their corresponding edge blocks share the same edge sign.

It remains to be shown that the obtained subgraph $G'$ is acyclic. We can directly conclude that $G'$ contains no triangles, since every triangle sign $\Delta _{(i,j,k)}$ has to be taken, prohibiting either (*i*, *j*), (*j*, *k*) or (*k*, *i*) (or two of them) to be part of $G'$. Assume that $G'$ contains a cycle $v_{i_1}, v_{i_2}, v_{i_3}, \ldots , v_{i_{l}}, v_{i_1}$ of length $l \ge 4$. Then, either $(v_{i_1},v_{i_3})$ or $(v_{i_3},v_{i_1})$ must be in $G'$. The latter would lead to a triangle, which we could already exclude from $G'$. But $(v_{i_1},v_{i_3}) \in G'$ implies that a circle of length $l-1$ also exists in $G'$. Repeated use of this argument implies that $G'$ also has a cycle with length 3, which is a contradiction to triangles being excluded. Thus, $G'$ cannot contain a cycle of length 4 or greater and must be acyclic.

In summary, the decision problem whether there is a solution for a dLOP instance (*G*, *k*) can be reduced to the decision problem whether a solution for the dLRS instance $(S, f_G(k))$ obtained from *G* exists. $\square$

## Solution strategies

To solve instances of LRS in practice we propose three reduction rules and two algorithmic approaches. As of Theorem 1 we cannot guarantee a polynomial running time.

### Reduction rules

In Sec. "Problem formulation" we already pointed out that an optimal solution for LRS always selects complete runs of characters and we reduced the notation of the input to runs of characters with a certain length each. This can also be seen as a reduction rule to the original problem formulation as the remaining size of the solution space now depends on the number of runs *n* instead of the actual string length *m*. Two more reduction rules rely on the following lemma:

#### Lemma 1

*Let* *S*, *T* *be two strings over the disjoint alphabets* $\Sigma _S$ *and* $\Sigma _T$. *Then the optimal LRS solutions for* *S* *and* *T* *can be concatenated to form an optimal solution for the concatenated string* *ST*.

#### *Proof*

Since the two alphabets are disjoint, an LRS solution for *S* does not contain any characters from $\Sigma _T$. Therefore the choice of the subsequence for *S* does not influence the valid subsequences for *T* and vice versa. This means that optimal solutions for *S* and *T* can be computed independently and concatenated to form a valid solution for *ST*. Obviously, an optimal solution for *ST* cannot be longer than the combined length of optimal solutions for *S* and *T*, otherwise the latter would not be optimal. $\square$

According to Lemma 1 we can divide an LRS instance *S* into smaller independent instances, if we find a prefix $r_1, \ldots , r_p$ of *S*, which uses an exclusive sub-alphabet $\Sigma '$, i.e., $r_1, \ldots , r_p \in {\Sigma '}^*$ and $r_{p+1}, \ldots , r_n \in \left( \Sigma \setminus {\Sigma '} \right) ^*$. This *prefix rule* can be applied in linear time by starting with the prefix $r_1$ and extending it until we either reach the end of *S*, in which case no independent suffix exists, or until the prefix is closed regarding the used characters. Let *p* be the index of the last occurrence of $\sigma (r_1)$. Since $\sigma (r_1)$ is used in the prefix, all runs $r_2, \ldots , r_p$ must belong to the prefix as well. Now start with $l=2$ and update *p* to the index of the last occurrence of $\sigma (r_l)$ (if this index is higher than *p*), increase *l* by 1 and repeat until $l > p$. If $p<n$, an independent prefix is found, otherwise such a prefix does not exist.

This idea can be extended to the *infix rule*, which finds independent infixes via the following lemma.

#### Lemma 2

*Let* *S*, *T* *be two strings over the disjoint alphabets* $\Sigma _S$ *and* $\Sigma _T$ *and let* *l* *be an arbitrary position in* *S*. *Then it holds that*

$$\begin{aligned} \text {LRS}\left( s_1 \ldots s_l T s_{l+1} \ldots s_m\right) = \text {LRS}\left( s_1 \ldots s_l \$^{\text {LRS}\left( T\right) } s_{l+1} \ldots s_m\right) \end{aligned}$$

*with* $$\$ \not \in \Sigma _S \cup \Sigma _T$$.

#### *Proof*

For the same reason as in Lemma 1 the instance *T* can be solved independently from *S*. For the combined string $s_1 \ldots s_l T s_{l+1} \ldots s_m$ the infix *T* is either entirely dropped in the optimal subsequence or the optimal solution of *T* itself is entirely taken as a part of the combined solution. Thus, *T* contributes either 0 or $\text {LRS}\left( T\right)$ characters to the optimal combined solution. Therefore, if the solution for *T* is already known, $s_1 \ldots s_l T s_{l+1} \ldots s_m$ can be solved by replacing *T* with a run of length $\text {LRS}\left( T\right)$ of a new character ${\$}$. $\square$

Following Lemma 2 we can search for an independent infix in *S* to obtain two smaller instances. Instead of starting with $r_1$, we start with an arbitrary character $\sigma \in \Sigma$ as anchor and use the infix $r_p, \ldots , r_q$ as a start with $r_p$ and $r_q$ being the first and last occurrence of $\sigma$, respectively. Similarly to the prefix search, we iterate over all runs in the infix and move the markers *p*, *q* to the left and right, whenever we encounter a new character with occurrences outside $r_p, \ldots , r_q$, until the infix is closed (with respect to used characters) or the entire string is contained. This is repeated with every character in $\Sigma$ as anchor, possibly yielding multiple infixes. Adjacent independent infixes are merged into larger ones, since we want as many runs as possible to be replaced with a single run. Infixes, which consist of only one run, are discarded, because they do not pose an actual reduction. Finding and merging all infixes can be done in time ${\mathcal {O}}(n \cdot |\Sigma |)$.

For a maximum reduction, the rules are applied as follows: First, the prefix rule is iteratively applied on *S* until no further independent prefix can be found. Second, the infix rule is applied on every sub-instance found so far. For every infix found the procedure is repeated by starting with the prefix rule again.

### Solving with integer linear programming

We present two algorithms to solve LRS to optimality, which have complementary strengths and weaknesses. The first is based on an Integer Linear Program (ILP). This approach scales well with large alphabets, but struggles with a large number of runs. We also propose a dynamic programming (DP) approach, which remains fast for long strings, but suffers from large alphabets. Both algorithms work exclusively on the runs of an input string *S*.

ILPs are a commonly used technique to model and solve combinatorial optimization problems. We model the LRS formulation from before as an ILP in the following way: Let *n* be the number of runs in *S* and let $x_1, \ldots , x_n$ be binary variables with $x_i = 1$ if $r_i$ is in the optimal subsequence and $x_i = 0$ otherwise. Any possible subsequence of runs can therefore be represented by a variable assignment. Since we want to maximize the length of the subsequence, we define our objective function as the weighted sum over all taken runs, using their lengths as weights. Let $r_i, r_j$ be two runs with $i<j$ and $\sigma (r_i) = \sigma (r_j)$. If both runs are selected, all intermediate runs $x_l$ with a different character must be excluded. This yields the following ILP:

4 $$\begin{aligned} \max \quad&\sum \limits _{i=1}^{n} x_i L(r_i) \end{aligned}$$

5 $$\begin{aligned} \text {subject to}\quad&x_l \le 2 - x_i - x_j&\forall \, i< l < j, \,\sigma (r_i) = \sigma (r_j) \ne \sigma (r_l) \end{aligned}$$

6 $$\begin{aligned}&x_i \in \{0, 1\}&\forall \,1 \le i \le n \end{aligned}$$

During the implementation it turned out that a single, more complex constraint for each pair $r_i, r_j$ with equal characters was solved slightly faster by the used ILP solver. Thus, we actually use the following equivalent set of constraints instead of (5):

7 $$\begin{aligned} \quad&\sum \limits _{\begin{array}{c} i<l<j\\ \sigma (r_l) \ne \sigma (r_i) \end{array}}x_l \le (j-i) \cdot (2 - x_i - x_j)&\forall \, i < j, \,\sigma (r_i) = \sigma (r_j) \end{aligned}$$

If either $r_i$ or $r_j$ are not taken, the respective constraint does not prevent any other combination of runs between them. The total number of constraints is bounded by ${\lceil \frac{n}{2} \rceil }^2$ and the number of non-zero entries in the constraint matrix is bounded by $n \cdot {\lceil \frac{n}{2} \rceil }^2$.

### Solving with dynamic programming

As an alternative to the ILP formulation the problem can also be solved bottom-up by a dynamic program (DP). Let *D*[*i*, *F*] be the length of an optimal LRS solution for $r_1 \ldots r_i$, which includes $r_i$ itself and only contains characters from $F \subseteq \Sigma$. The DP can be initialized with $D[0, \emptyset ] = 0$ and $D[0, F] = -\infty$ for $F \ne \emptyset$. Known solutions can be extended run by run, always selecting an optimal predecessor for each run and keeping track of already used characters with the second parameter *F*. For $\$ \not \in \Sigma$, let $R_S(i) = \left\{ P_{\sigma }(i) \mid \sigma \in \Sigma \cup \{\$\}, P_{\sigma }(i) \ge P_{\sigma (r_i)}(i) \right\}$ contain the positions of the last occurences for every $\sigma \in \Sigma$, between position *i* and the last occurence of $\sigma (r_i)$ before *i* (or 0 if *i* is the first occurence of its kind). If $r_i, r_j$ are two consecutive runs of an optimal solution, there can be no other runs between *i* and *j* using the same character, as this would make the solution sub-optimal. Thus, if an optimal solution contains a run $r_i$, it either is the first selected run or the predecessing run must be from a position $j \in R_S(i)$. This restricts the number of possible predecessors for each run in the DP by ${\mathcal {O}}(|\sigma |)$. The full DP is then as follows:

8 $$\begin{aligned} D[0, \emptyset ]&= 0 \end{aligned}$$

9 $$\begin{aligned} D[0, F]&= -\infty \qquad \forall F \ne \emptyset \nonumber \\ D[i, F]&= \max \limits _{j \in R_S(i)}\left\{ \begin{array}{ll} D[j, F] + L(r_i) &{} \text {if } \sigma (r_j) = \sigma (r_i) \\ D[j, F\setminus \{\sigma (r_i)\}] + L(r_i), &{} \text {if } \sigma (r_j) \ne \sigma (r_i) \end{array}\right\} \end{aligned}$$

**Fig. 3:**
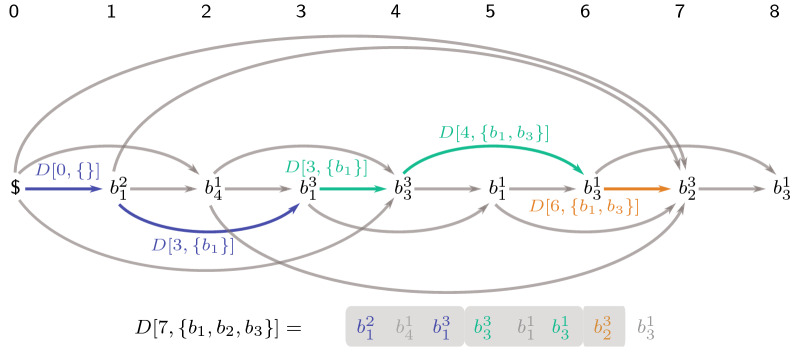
Graph visualizing the recursion for the running example. Arcs represent the possible predecessors for every run. Colors mark an optimal path and the DP entries taken by the recursion

The recursion can be visualized by a directed acyclic graph as shown in Fig. 3. It contains a start vertex corresponding to the empty prefix of *S* and one vertex for every run in *S*. Every path in the graph corresponds to a (possibly invalid for LRS) subsequence of *S*. Each vertex *i* has an incoming edge from each position $j \in R_S(i).$

*D*[*i*, *F*] is computed by taking all possible predecessor positions *j* and extending the solutions by $r_i$. If $\sigma (r_i) = \sigma (r_j)$, the solution is extended by $r_i$ without introducing a new character. The length for the new solution would be the optimal length for positon *j*, using the same sub-alphabet *F* and adding the length of $r_i$. For $\sigma (r_i) \ne \sigma (r_j)$ the used sub-alphabet must also be extended by $\sigma (r_i)$, requiring to look up the previous solution from $D[i, F\setminus \{\sigma (r_i)\}]$ instead of *D*[*i*, *F*].

An optimal solution for LRS can be found by taking the entry of *D* with the highest length and using the backtracking information from the DP to obtain the corresponding subsequence. The DP table has a total of $n+1$ columns and $2^{|\Sigma |}$ rows with each entry taking ${\mathcal {O}}(|\Sigma |)$ time to compute. This leads to a worst-case runtime of ${\mathcal {O}}\left( |\Sigma | \cdot n \cdot 2^{|\Sigma |}\right)$ for the DP, making this a *fixed parameter tractable* (FPT) approach for LRS with the alphabet size as parameter.

## Experiments

We performed computational experiments on two different types of instances. First, we generated random instances to see how the two algorithms scale on string length and alphabet size. Second, we integrated the algorithms into the software SyRI [7], which finds structural rearrangements between two assemblies of related species and has an additional stage for homology-based scaffolding, where the algorithms are used.

The ILP has been implemented using the Python interface of PuLP, which solves the ILP with the free solver CoinOR.^1^ All tests were run on an AMD Epyc 7742 processor with 1TB of memory running on Debian. The algorithms are implemented in Python and executed via Snakemake [10] using Python 3.9.1 and PuLP version 2.3.1.

### Synthetic data

The synthetic data was created by randomly generating strings with different lengths and alphabet sizes. For any combination a total of 20 strings was generated, such that every string is guaranteed to use the entire alphabet assigned to it. These instances pose worst-case instances for our algorithms, as the proposed reduction rules can hardly be applied. The runs are quite short in general and since there is no structurally induced locality among the characters, instances could be split very rarely. All instances were solved with all reductions rules applied.

**Fig. 4:**
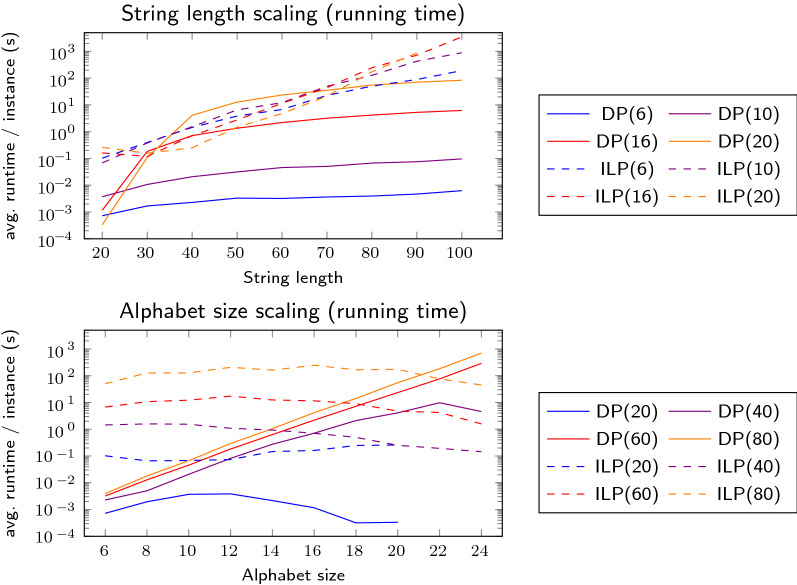
Running time plotted against string length (top) and alphabet size (bottom). Each curve represents an algorithm and an additional parameter (number in parentheses), which is alphabet size in the top plot and the string length in the bottom plot

Figure 4 shows how the runtime scales with both increasing string lengths and increasing alphabet size. For a fixed alphabet size the runtime scales about exponentially with the string length for the ILP as shown in the top plot. In fact, the alphabet size only has very minor effect on the ILP compared to the string length, which becomes visible in the bottom plot, with a slight favor of larger alphabets. The DP behaves complementary to the ILP, scaling exponentially in the alphabet size and sub-exponentially with string length.

**Fig. 5:**
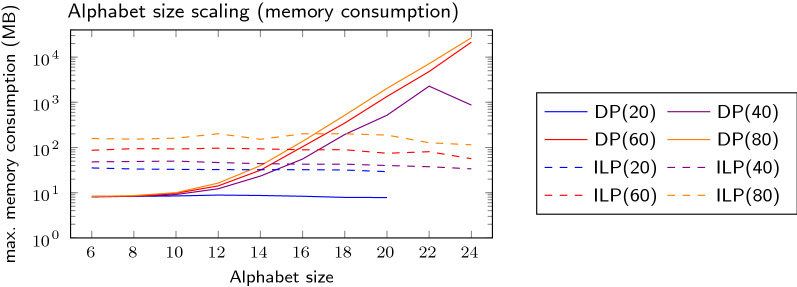
Memory consumption plotted against alphabet size. Each curve represents a combination of an algorithm a string length, printed in parentheses

The scaling can be explained by the properties of the algorithms. The ILP has a binary decision variable for every run, increasing the number of possible (but not necessarily feasible) variable assignments exponentially with the number of runs. Once the ILP solver has to fall back to branch-and-bound, the scaling becomes exponential. Larger alphabets might lead to a lower number of constraints (and thus a lower runtime), as the ILP contains one constraint for every pair of runs with the same character. As already pointed out in Sect.﻿ "Solving with dynamic programming" the DP table grows linearly with the number of runs and exponentially with alphabet size. This is reflected both in running time and memory consumption shown in Fig. 5. Especially the latter is problematic, as alphabet sizes of 24 or higher might require more memory than a usual desktop computer offers. The ILP consumes more memory than the DP on small alphabets, but shows no increased memory footprint as the alphabet size grows. The decreasing running time for very large alphabets is caused by the reduction rules, as it leads to a higher number of characters occurring only in a single run and thus to a higher chance of the string being splittable into independent parts.

Based on this empirical data, the final version of our tool uses both algorithms depending on string length and alphabet size. If $|s | < 10 (|\Sigma |-13)$ the ILP is preferred, otherwise it is the DP.

### Biological data

The LRS model is being used to generate homology-based pseudo-chromosome level assemblies in the chroder method of SyRI [7], i.e., the process of creating homology-based chromosome-level assemblies in case only scaffold-level assemblies are available. We consider a dataset which was generated in [7] to test the performance of an approach to find structural rearrangements. It consists of 100 fragmented assemblies of varying contiguity that have been generated by introducing 10 to 400 random breaks in chromosome-level assemblies of the Col-0 and L*er* accessions of *Arabidopsis thaliana* [7, 11]. We used the LRS-based chroder method to scaffold these assemblies in order to estimate the usefulness of the model in generating homology-based pseudo-chromosomes. The LRS instances were created by mapping both sets of contigs against each other using nucmer [12] and dividing the contigs into equally long bins afterwards. For each bin the best matching contig of the other contig set is determined based on the previously computed local mappings of each contig. Considering that each bin can only be assigned to one contig and represents one character in the constructed LRS instance, shorter bins preserve more information, but also increase the complexity of the LRS instance. The bin size was empirically chosen as 10kb producing reasonable results while maintaining solvable instances. The scripts and used assemblies are made publicly available.

**Fig. 6:**
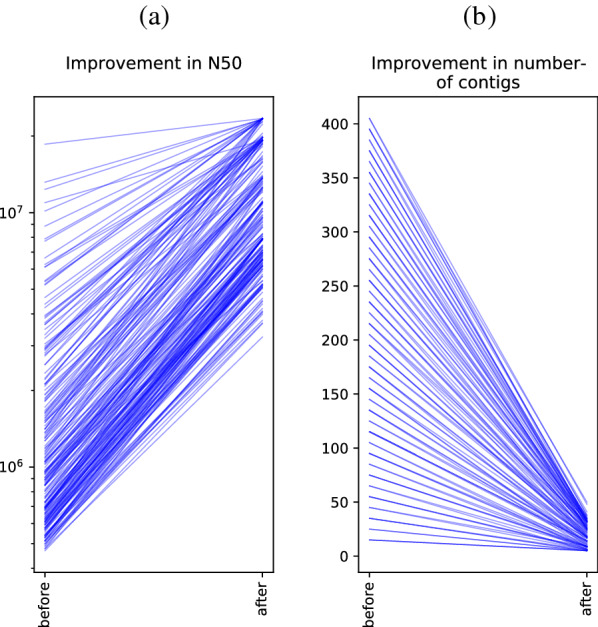
Performance improvement of using the homology-based scaffolding of chroder. **a** Increase of N50 values between raw (scaffold-level) assemblies and the output of chroder using LRS. Each line represents one of 100 generated fragmented assemblies. **b** Decrease in contig count between both assemblies

For more than 85% of the simulated genomes (both Col-0 and L*er*) the pseudo-chromosome N50 values resulting from solving the LRS problem within chroder were five times higher than those of the corresponding fragmented assemblies, with the N50 being even more than ten times higher for more than 30% of the samples (see Figure 6a). For many of the highly fragmented assemblies it is difficult to order fragments because of the presence of repetitive regions in both genomes. Note that, even if the LRS-based method is not able to generate the original full length genomes in these cases, it significantly decreases the number of disjoint fragments, increasing assembly contiguity as shown in Figure 6b.

Originally the ordering problem in chroder was solved using a brute-force method, which was unable to solve 16 out of 100 instances within a reasonable amount of time and memory. We tested these 16 LRS instances separately and ran them using the DP and ILP algorithms presented in this paper.

Using all three reduction rules, both algorithms were able to solve all instances in very short computation time, thus demonstrating the practical efficiency of our algorithms, see Table 1. For this reason, the previous brute-force method has been replaced by the implementation of our algorithms within the chroder script of the SyRI package. In fact, the instances were almost completely solved by the prefix rule alone, resulting in many trivial sub-instances (singleton runs). Note that for these singleton runs we did not call the ILP solver as the overhead of setting up the ILP would have dominated the running time. However, not using the prefix or infix rule reveals differences between both algorithms. The alphabet sizes between 31 and 38 caused the DP to run out of memory, while the ILP remained fast with the longest instance consisting of only 50 runs.

**Table 1:** Comparison of runtime (in seconds) between DP and ILP on instances from real data. The times are for all 16 instances that proved difficult for the previous brute-force method. The columns correspond to different reduction rules used

Algorithm	All rules	No infix rule	No prefix and infix rule
DP	0.006	0.003	Out of memory
ILP	0.006	0.006	0.56

## Discussion

Note that the purpose of this paper is not to present a full novel scaffolding method but rather to introduce an algorithm that may prove useful in existing methods for scaffolding. We demonstrated its usefulness within the first phase of the SyRI tool that needs chromosome-level assemblies as input.

The experiments showed that optimal LRS solutions can be found in short time for instance sizes that occur on assemblies of real samples. We presented two different algorithms whose running times depend on two important instance properties, namely string length and alphabet size. Random strings, however, do not seem to resemble actual assembly instances, which are already pre-sorted except for some noise or rearrangements. The reduction rules have little to no impact on random strings, while they reduce the assembly instances to almost trivial sub-instances. This implies that reduction rules might be more important in practice than the algorithm to process the remaining preprocessed instance.

One potential problem of the model itself was mentioned in Sect. "Problem formulation". LRS only allows for one run per character, which automatically induces an ordering on the underlying contigs. This can be problematic if the binned contig contains a translocation that splits a long run into two, e.g., $b_1 b_1 b_1 b_2 b_2 b_2 b_1 b_1 b_1$. The LRS model will drop one of the $b_1$ runs, even though it would be better to leave the order of $B_1$ and $B_2$ open due to lack of evidence.

Another limitation arises while mapping the bins. Since only the best match for every bin is taken, any mapping ambiguity is ignored, which might drop valuable information. There is also no support for inversions inside the model. While inverted alignments can be taken into account for the mapping step of a single bin, the model stays unaware of inversions and the fact that an interval of bins is actually in the reverse order compared to the second assembly. However, this might not be as problematic as it sounds, because the bins are not mapped to other bins but to entire contigs. As long as inversions are contained in a single contig, they should have no impact on the ordering that the model produces.

## Conclusion

Ordering contigs by means of an incomplete assembly of a related species occurs as a variant of homology-assisted assembly, which does not require chromosome-level assemblies already. We introduced the Longest Run Subsequence (LRS) problem, formalizing the contig ordering problem as a string problem. We proved that LRS is NP-hard and presented reduction rules and two algorithms, which work well for long instances and large alphabets, respectively, which we showed on a synthetic data set. Regarding real data, we managed to solve all instances that could not be solved by a brute force approach in short computation time. In fact, the original brute-force-based method in the popular SyRI tool has been replaced by the open-source implementation of our algorithms.

From the theoretical side, we find it interesting to further investigate approximability and fixed-parameter tractability of LRS. Some of these suggestions have been picked up in a recent preprint [13]. From a practical perspective, we plan to further test the approach on real assembly data, also taking more than two related assemblies into account.

## Data Availability

The source code and snakemake pipeline to create and run the simulated data is available at https://github.com/AlBi-HHU/longest-run-subsequence. The software itself can be installed from https://pypi.org/project/longestrunsubsequence/. A collection of all used code and data, including the experiments which two assemblies from Arabidopsis thaliana have been uploaded to Zenodo at 10.5281/zenodo.4552211.

## Footnotes

1 https://github.com/coin-or/pulp.
